# Acceleration of *Enterococcus faecalis* Biofilm Formation by Aggregation Substance Expression in an *Ex Vivo* Model of Cardiac Valve Colonization

**DOI:** 10.1371/journal.pone.0015798

**Published:** 2010-12-30

**Authors:** Olivia N. Chuang-Smith, Carol L. Wells, Michelle J. Henry-Stanley, Gary M. Dunny

**Affiliations:** 1 Department of Microbiology, University of Minnesota Medical School, Minneapolis, Minnesota, United States of America; 2 Department of Laboratory Medicine and Pathology, University of Minnesota Medical School, Minneapolis, Minnesota, United States of America; Loyola University Medical Center, United States of America

## Abstract

Infectious endocarditis involves formation of a microbial biofilm *in vivo*. *Enterococcus faecalis* Aggregation Substance (Asc10) protein enhances the severity of experimental endocarditis, where it has been implicated in formation of large vegetations and in microbial persistence during infection. In the current study, we developed an *ex vivo* porcine heart valve adherence model to study the initial interactions between Asc10^+^ and Asc10^−^ *E. faecalis* and valve tissue, and to examine formation of *E. faecalis* biofilms on a relevant tissue surface. Scanning electron microscopy of the infected valve tissue provided evidence for biofilm formation, including growing masses of bacterial cells and the increasing presence of exopolymeric matrix over time; accumulation of adherent biofilm populations on the cardiac valve surfaces during the first 2–4 h of incubation was over 10-fold higher than was observed on abiotic membranes incubated in the same culture medium. Asc10 expression accelerated biofilm formation via aggregation between *E. faecalis* cells; the results also suggested that *in vivo* adherence to host tissue and biofilm development by *E. faecalis* can proceed by Asc10-dependent or Asc10-independent pathways. Mutations in either of two Asc10 subdomains previously implicated in endocarditis virulence reduced levels of adherent bacterial populations in the *ex vivo* system. Interference with the molecular interactions involved in adherence and initiation of biofilm development *in vivo* with specific inhibitory compounds could lead to more effective treatment of infectious endocarditis.

## Introduction

*Enterococcus faecalis* is a gram-positive bacterium that normally resides in the gastrointestinal tract of humans. However, this microbe is also capable of causing disease, as it is responsible for infections such as infectious endocarditis, urinary tract and wound infections, and bacteremia [1], [2]. It is a naturally antibiotic-resistant and tenacious organism that survives harsh conditions such temperatures as high as 60°C, and in tap water [3], making it difficult to eliminate as an opportunistic pathogen.

Enterococci are the third leading cause of infectious endocarditis, accounting for 20% of all bacterial endocarditis cases. The hallmark of this disease is the vegetation, a clotted mass composed of platelets, fibrin, large numbers of bacteria, and immune cells. Two routes of vegetation formation have been described: (1) non-bacterial thrombotic vegetation formation, and (2) vegetation formation on previously undamaged, healthy valves. In the first case, valvular damage is inflicted by events such as valvular regurgitation which results in irregularities in blood flow, or scarring on valve tissue due to prolonged intravenous drug use [4]. These lead to damage of the valve tissue, which in turn recruits platelets and fibrin to the damaged site. Once bacteria enter the bloodstream, whether through routes such as during surgical procedures or translocation through the intestinal tract, then the sterile vegetation of platelets and fibrin is infected with these bacteria to become the septic vegetation [5].

In the second case, no previous valve aberrancies are noted in the patient, but some types of bacteria, once in the bloodstream, can inflict damage on the endothelial cell layer of the valve tissue [6], [7]. This route of vegetation formation has been proposed for *Staphylococcus aureus*, where the bacterium is endocytosed by valve endothelial cells, and can trigger initiation of the coagulation cascade. Eventually, the bacterial cells lyse the endothelial cells, which amplifies the coagulation cascade, bringing more platelets and fibrin to bind to the damaged site [8]. *E. faecalis* has been noted in some cases to cause endocarditis in patients who have no past history of valve aberrancies, so this route of vegetation formation also could be applicable to *E. faecalis* [7].

The vegetation itself is considered a biofilm, with bacteria encased inside layers of fibrin and platelets, and other pro-coagulant factors; growth in this environment impedes the effectiveness of antibiotic and immune mediated microbial killing [9]. Though antibiotics are able to enter the vegetation, the physiological state of the bacteria in the biofilm could prevent the antibiotics from damaging the bacteria [10]. Durack *et al.* demonstrated that where bacteria are not protected by fibrin layers, macrophages were able to phagocytose the exposed bacteria [11]. In addition, antibodies against Asc10 were unable to penetrate the established vegetation to opsonize the *E. faecalis* cells [12].

**Figure 1:**
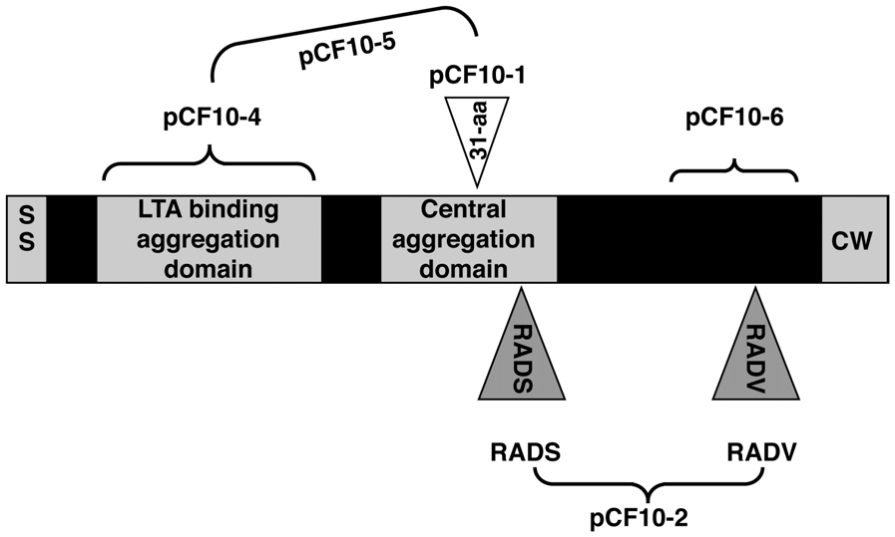
Asc10 variants used in this study; *prgB* mutant constructs were generated in the native context of pCF10. Wild-type Asc10 protein shown in shaded areas. Derivatives of pCF10 are shown in different regions; brackets denote altered regions of protein. pCF10-1, 31-aa insertion in central aggregation domain at aa 546; the central aggregation domain was postulated to fall between aa 473–683 in previous studies [15], but it is possible that it covers a larger region. pCF10-2, RGD motifs changed to RAD (the more N-terminal RGDS motif falls at aa 606, RGDV at aa 939). pCF10-4, deletion of N-terminal lipoteichoic acid (LTA) binding aggregation domain (aa 156–358); pCF10-5, combined mutations in both aggregation domains; pCF10-6, C-terminal domain deletion (aa 688–1138). pCF10-8 is not shown here, but this mutant derivative is contains an in-frame deletion of the *prgB* gene, which retains only the first three and last three codons.

To avoid difficulties in eliminating the bacteria once they become established in this type of biofilm, it would be ideal to block initial bacterial adherence or initial biofilm development in the nascent vegetation. For *Enterococcus faecalis*, aggregation substance (Asc10) is one of several known adhesins that could be targeted for treatment against infectious endocarditis. Asc10 is a 137-kDa surface protein expressed from the *prgB* gene on the pCF10 conjugative plasmid; its expression in donor cells is triggered during conjugation by a recipient-produced pheromone. Asc10 expression on the donor cell surface promotes binding to recipient cells. Expression of the remaining pCF10-encoded conjugation machinery follows in the donor cell, with subsequent transfer of plasmid DNA. Asc10 can be expressed *in vivo*, in the absence of recipient cells, and cells carrying pCF10 have a selective advantage in an endocarditis model over those without this plasmid [13], [14]. Several domains in Asc10 have been previously identified and characterized [15], [16], including two aggregation domains that are required for donor-recipient binding, and two RGD motifs that play an important role in endocarditis pathogenesis, possibly involving immune evasion (**Fig. 1**).

McCormick *et al.* [17] demonstrated that antibodies against an N-terminal fragment of Asc10 (encompassing aa 44–331) were not protective in the rabbit model of endocarditis. In contrast, Fab fragments purified from polyclonal antibodies against full-length Asc10 protein were able to decrease severity of endocarditis in rabbits [18]. It is likely that whole antibodies against Asc10 promote increased aggregation between Asc10-expressing cells. These aggregated masses of bacteria are then trapped in small passages of the lungs, where lung congestion has been observed and likely contributes to the death of the host. In addition, the increased aggregation leads to formation of larger vegetations. In animals immunized passively with Fab fragments, the antigen-binding arms of the antibody are not linked by the Fc portion, and thus aggregation between bacterial cells is reduced. These Fab fragments may also block interactions of Asc10 with host immune cells, which could promote *E. faecalis* persistence. Thus Asc10 is a potential target for antimicrobials against endocarditis.

Our past studies using a rabbit endocarditis model [16], suggested roles of Asc10 in colonization of vegetations and in resistance to the host immune response. We sought to dissect further the process of vegetation formation by focusing on the initial interactions between the bacteria and the valve tissue. In the current study, we developed an *ex vivo* porcine heart valve adherence assay to study these interactions. We also employed the system to examine early biofilm development on a relevant host surface. The results show that Asc10 expression accelerates biofilm development and that adherence and biofilm development by both Asc10^+^ and Asc10^−^ strains is markedly increased on cardiac valve surfaces, relative to abiotic membranes.

## Results

### Adherence and biofilm formation on porcine heart valves

**Figure 2:**
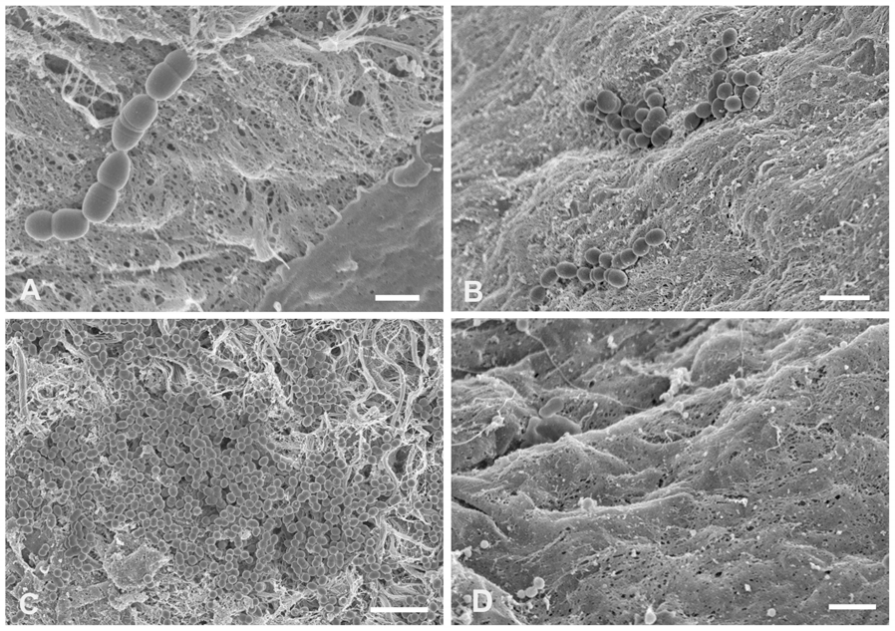
Increase in Asc10^+^ *E. faecalis* aggregate size on valve tissue over time. Scanning electron micrographs of *E. faecalis* Asc10^+^ OG1SSp (pCF10) incubated with heart valve segments for 0.5 h (A), 2 h (B), and 4 h (C), showing noticeable enlargement of bacterial aggregates over time, an observation compatible with biofilm formation. Part A also highlights preferential adherence of *E. faecalis* to areas of noticeable tissue damage, as opposed to areas where the tissue appears more intact (lower right of photograph). An uninfected valve is shown in part D, where the tissue is mostly intact, compared to the other panels in the presence of bacteria. Scale bars: A, 1 µm; B, 2 µm; C, 4 µm; D, 1 µm.

Endocarditis likely initiates with adherence of bacteria from the bloodstream to the valve surface, followed by their growth as a biofilm. As a simple model for the early stages of endocarditis, we incubated excised porcine heart valve sections in endothelial cell medium, (ECM; Materials and Methods) with isogenic strains of *E. faecalis* carrying wild-type pCF10 or mutant derivatives, and examined numbers and surface distribution of the adherent bacterial populations by plate counts and by high resolution field-emission scanning electron microscopy (FESEM). We expected that at incubations shorter than 1 h, adherence would be the primary contributor to the population increase, whereas surface growth would become a more important contributor at later time points. The liquid medium containing each valve section was inoculated with 10^6^ CFU/ml of the bacterial strain being tested to start each experiment. For all strains, the bacterial population density of the liquid phase increased about 100 fold during the 4 h duration of the experiment with 30–35 min generation times in the planktonic phase (not shown). We restricted the duration of our experiments to 4 h to focus on adherence and early biofilm development under conditions where the growth medium was not depleted. We initially carried out FESEM analysis of the time course of surface colonization by the Asc10^+^ strain OG1SSp (pCF10). Representative fields are shown in Figure 2. Adherent bacteria were readily visualized within 30 minutes of incubation, and both the total numbers and the size of the attached multicellular bacterial aggregates increased remarkably during the 4 h time course. After 30 minutes, most of the attached bacteria were in short chains (Fig. 2A) or diplococci. After 2 h of incubation, we observed many attached small microcolonies in the range of 10–50 cells (Fig. 2B), and by 4 hours groups containing hundreds of cells were prominent (Fig. 2C). The valve surfaces contained areas of obvious physical damage to the surface layer of endothelial cells, as well as areas where the surface appeared intact, as seen in Fig. 2A (contrast lower right-hand corner to rest of tissue) and Fig. 2D (uninfected control valve). The bacteria frequently adhered to the damaged areas; this may mimic the conditions in the rabbit model where a catheterization procedure prior to infection is used to damage the valve surface in vivo [12], [16], [19] and facilitate subsequent bacterial colonization.

### Contribution of Asc10 to adherence and biofilm formation

**Figure 3:**
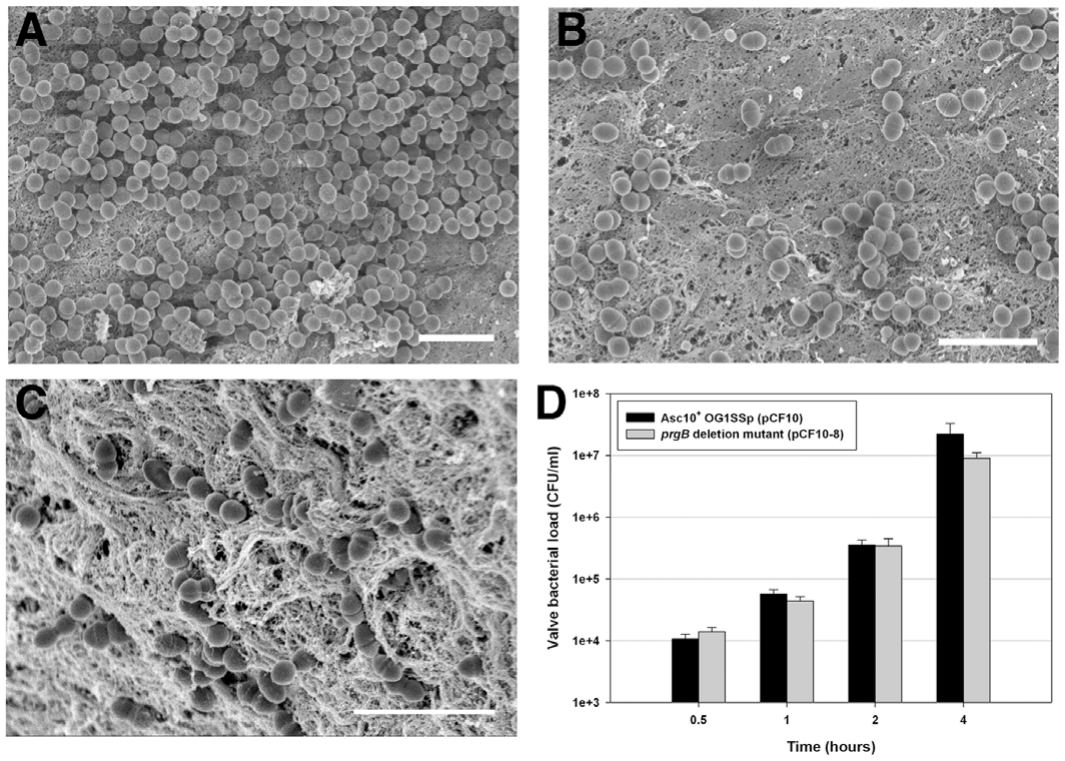
Asc10^+^ OG1SSp (pCF10) colonizes valve tissue more heavily than an Asc10^−^ *prgB* deletion mutant. (**A, B, C**) Valve tissue infected with Asc10^+^ OG1SSp (pCF10) (panel A, B) and Asc10^−^ *prgB* deletion mutant strains (OG1SSp [pCF10-8]; C), as analyzed by scanning electron microscopy. Note the density of Asc10^+^ OG1SSp (pCF10) cells colonizing the valve (A), though not all areas of the valve were colonized as heavily, as shown in part B. In contrast, the *prgB* deletion mutant bound in single cells or short chains; all images were taken at 4 h post-infection. Scale bar: A, B, C  = 3 µm. (**D**) Porcine aortic, tricuspid, and mitral valves were infected with *E. faecalis* strains carrying wild-type pCF10 and the *prgB* deletion derivative pCF10-8 for 0.5, 1.0, 2.0 and 4.0 h. Valves were washed and homogenized, and adherent bacteria were quantified by plating onto agar. The data shown are a compilation of at least three experiments, each with valve sections from a different heart.

Having confirmed the robust ability of *E. faecalis* to colonize the heart valves as multicellular microcolonies detectable by FESEM, we examined the specific contribution of Asc10 to this process by comparative examination of isogenic strains where the only genetic difference was whether the strain expressed an intact *prgB* gene or an in-frame deletion allele. The results are shown in Figure 3. Comparison of Fig. 3A and B versus 3C illustrates that significant numbers of both strains were able to colonize the valve surface during a 4 h incubation period, but the Asc10^+^ strain generally formed large attached aggregates (Figs. 3A & 2C), while most of the adherent Asc10^−^ organisms were distributed on the surface as short chains (Fig. 3C). Though the Asc10^+^ strain was commonly found in aggregates, there were also areas that were colonized less heavily (Fig. 3B). When we enumerated the adherent populations following their physical removal from the tissue surface, we observed similar colonization by both strains for the first 2 h, while at 4 h, the Asc10^−^ strain colonized at about 40% of the level observed with the Asc10^+^ strain (Fig. 3D). We think that this difference is biologically significant as the enumerations of adherent bacteria reported here are cumulative results of 3 or more separate experiments done on different days with valves from different hearts, and we carefully normalized the volumes of liquid medium and buffers used in the experiments to the mass of the valve sections used in each experiment (Materials and Methods). Note also that the difference of 1.31×10^7^ CFU/ml between the bacterial load of the Asc10^+^ (2.22×10^7^ CFU/ml) versus Asc10^−^ (9.09×10^6^ CFU/ml) strains could be readily observed by SEM (cf. Figs. 3A and 3C). In addition, we found that use of either sonication, pestle treatment, or EDTA (known to disperse Asc10-mediated bacterial clumping [20]) for removal and resuspension of the bacteria attached to the valves resulted in similar numbers of CFU on the enumeration plates from the assays carried out with Asc10^+^ strains (see Supporting Information: Figure S1). This indicated that re-aggregation during plating did not have a significant effect on the enumeration of the attached Asc10^+^ strains.

**Figure 4:**
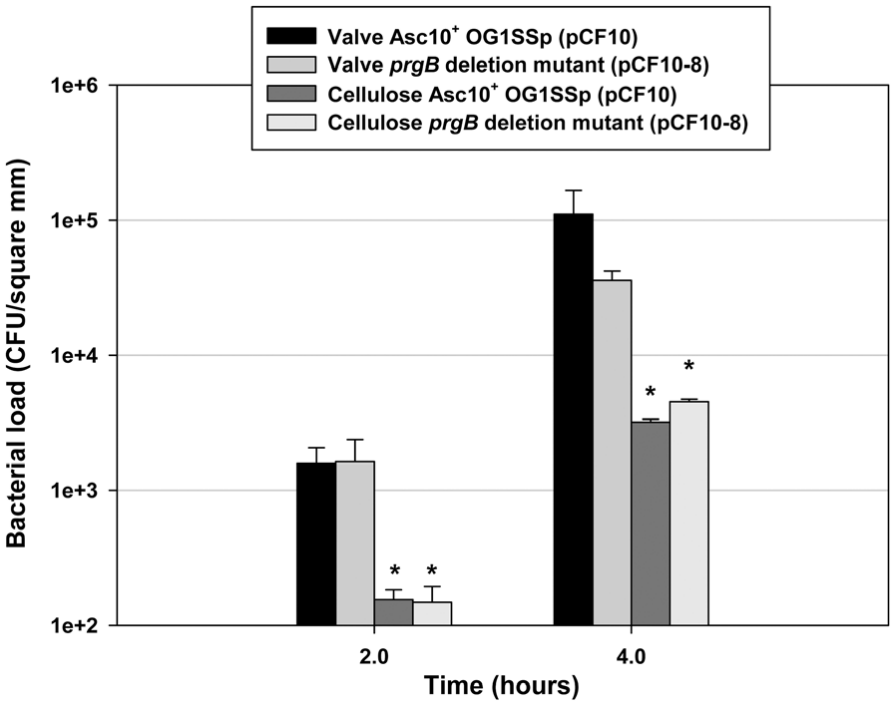
Comparison of porcine heart valve tissue and cellulose membrane adherence by Asc10^+^ OG1SSp (pCF10) and a *prgB* deletion mutant (pCF10-8). Both strains are demonstrated to bind to valve tissue at a much higher density than to an abiotic surface such as the cellulose membrane. * indicates p-value ≤0.02, with respect to valve bacterial load.

Our previous studies have employed submerged dialysis membranes as abiotic surfaces for biofilm development in vitro [21], [22]. While these surfaces support formation of robust biofilms with extensive 3-dimensional cellular structure and production of extracellular matrix [22], the accumulation of adherent enterococcal populations on the heart tissue is much more rapid than with the synthetic membranes. As shown in Figure 4, in the 2–4 h time frame, the valve surfaces became colonized with about 10-fold higher bacterial loads/surface area than observed with the membranes; similar results were obtained with a second type of synthetic membrane (Aclar) that also supports enterococcal biofilm development (not shown). Increased biofilm formation on cardiac valves relative to the membranes was observed with both Asc10^+^ and Asc10^−^ bacteria, with a larger effect observed at 4 h for the Asc10^+^ strain.

**Figure 5:**
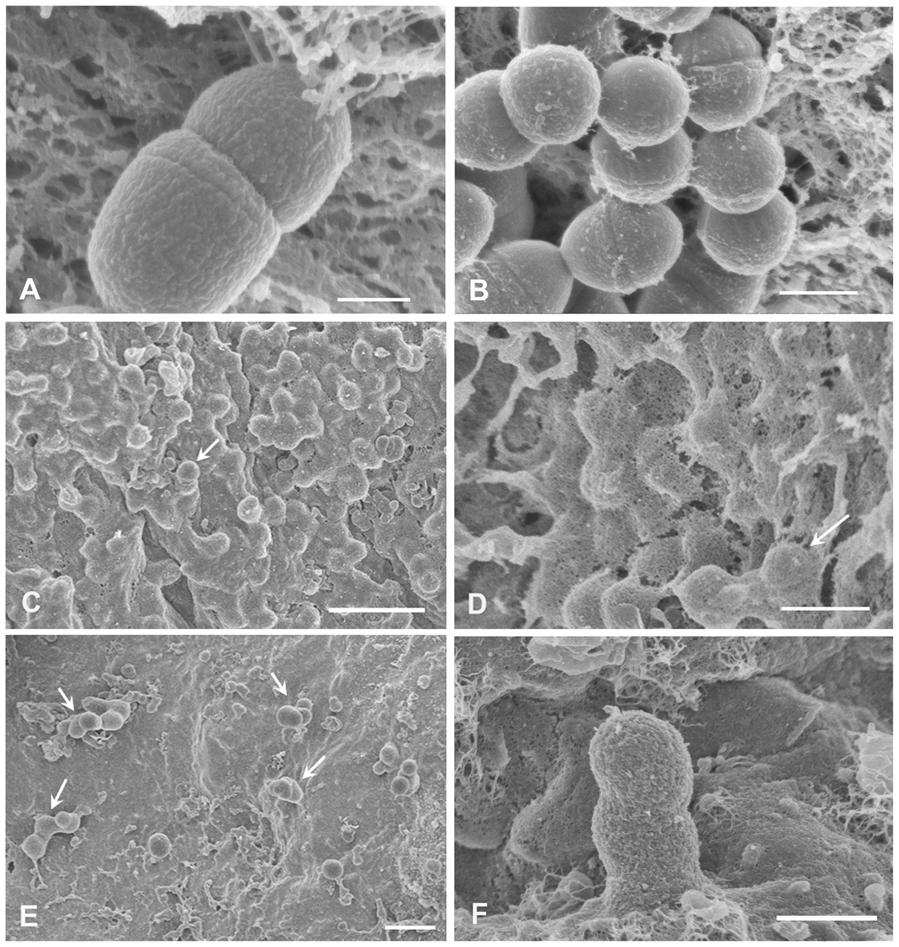
Greater accumulation of exopolymeric matrix on Asc10^+^ *E. faecalis* biofilms, in comparison to Asc10^−^ biofilms. Scanning electron micrographs of *E. faecalis* Asc10^+^ OG1SSp (pCF10) incubated 0.5 h (**A**) and 2 h (**B**) showing typical comparatively smooth appearance of the bacterial surface at early incubation times (**A**) compared to the fibrillar strands that became more evident over time (**B**). Use of a fixative containing alcian blue on the 4 h Asc10^+^ OG1SSp (pCF10)-infected valve tissue reveals the presence of an interwoven matrix covering most of the bacterial cells, which are seen as raised areas under the matrix (**C**). At higher magnification (**D**), bacterial cells can be observed clearly under the matrix, which appears more fibrillar in this view. Arrows in parts C and D indicate cells partially covered by matrix material. (**E, F**) Alcian blue-fixed valve sections colonized with the *prgB* deletion mutant (pCF10-8) revealed less matrix material as compared to the Asc10^+^ strain, in addition to a marked decrease in bacterial cells adherent to the valve tissue. In part F, the matrix material coats an Asc10^−^ *E. faecalis* cell, with areas of attachment to the valve tissue. Scale bars: A = 0.3 µm, B = 0.5 µm, C = 3.0 µm, D = 1.0 µm, E = 4.0 µm, F = 1.0 µm.

In addition to the formation of multicellular microcolonies, biofilm formation typically involves synthesis of an extracellular matrix that serves to physically stabilize the adherent community. We have shown that *E. faecalis* biofilms formed on abiotic surfaces produce matrix material that can be visualized by FESEM [22]. This matrix can appear as linear fibrillar material that connects the bacterial cells to the surface and to one another, or as a dense, interwoven matrix depending on the age of the biofilm and other conditions. Treatment with the cationic dye alcian blue enhances visualization of the interwoven matrix by SEM, indicating that it may contain anionic polysaccharides [22], [23]. Figure 5A shows an attached cell after 30 minutes of incubation, whose surface is generally devoid of extracellular material, while Fig. 5B shows some extracellular fibrillar material that was observed after 2 h (without alcian blue treatment). By 4 h of surface growth a dense matrix coating the Asc10^+^ biofilm cells was detected in alcian blue-treated samples (Figs. 5C and 5D). As described above (Fig. 3C), attached Asc10^−^ microcolonies were comprised of much smaller groups of cells and alcian blue treatment of these samples revealed less of the interwoven matrix material (Fig. 5E), although this material was still detectable in the matrix formed by Asc10^−^ cells, especially at higher magnification, and this material also appeared to attach the bacteria to the valve tissue surface (Fig. 5F). We conclude that the attached cells observed in these experiments are undergoing a biofilm developmental process that is enhanced when Asc10 is expressed. Asc10 is not readily visualized with this type of SEM imaging unless immunogold labeling is used [24], so the extracellular material observed is not likely Asc10 itself, but matrix material whose synthesis may be increased by Asc10-mediated enhancement of biofilm formation.

### Contribution of Asc10 subdomains to adherence and biofilm development

**Figure 6:**
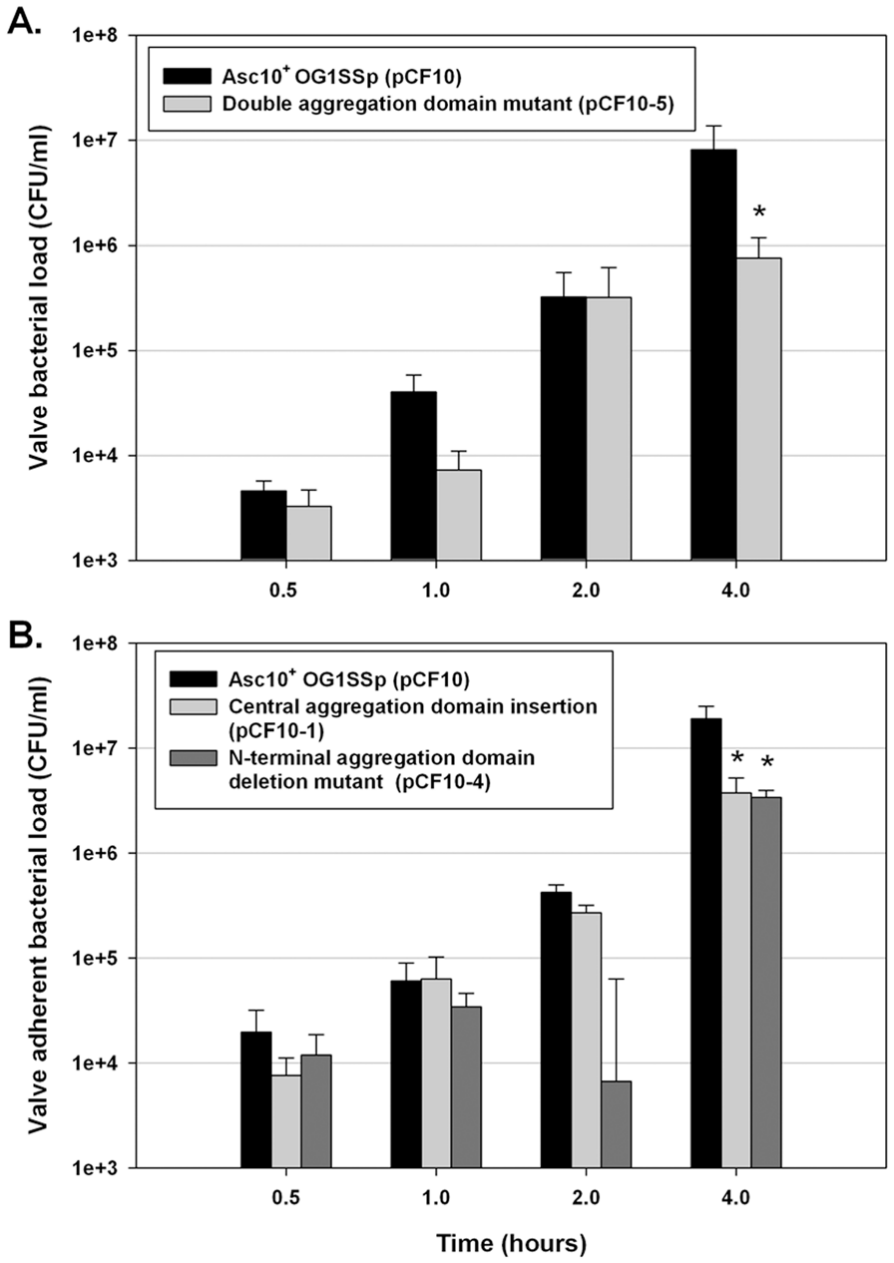
Alteration of the aggregation domain mutants in Asc10 lowers the ability of *E. faecalis* to bind to valve tissue. (**A**) Double aggregation domain mutant (OG1SSp [pCF10-5]) with N-terminal aggregation domain deletion and central aggregation 31-aa insertion. The mutant is unable to bind as well as Asc10^+^ OG1SSp (pCF10). (**B**) Single aggregation domain mutants bind as well as Asc10^+^ OG1SSp (pCF10) initially, but over time the gap between the Asc10^+^ OG1SSp (pCF10) and mutant grows increasingly. The data shown are a compilation of at least three experiments. * denotes p≤0.02, with respect to Asc10^+^ OG1SSp (pCF10) valve bacterial loads.

**Figure 7:**
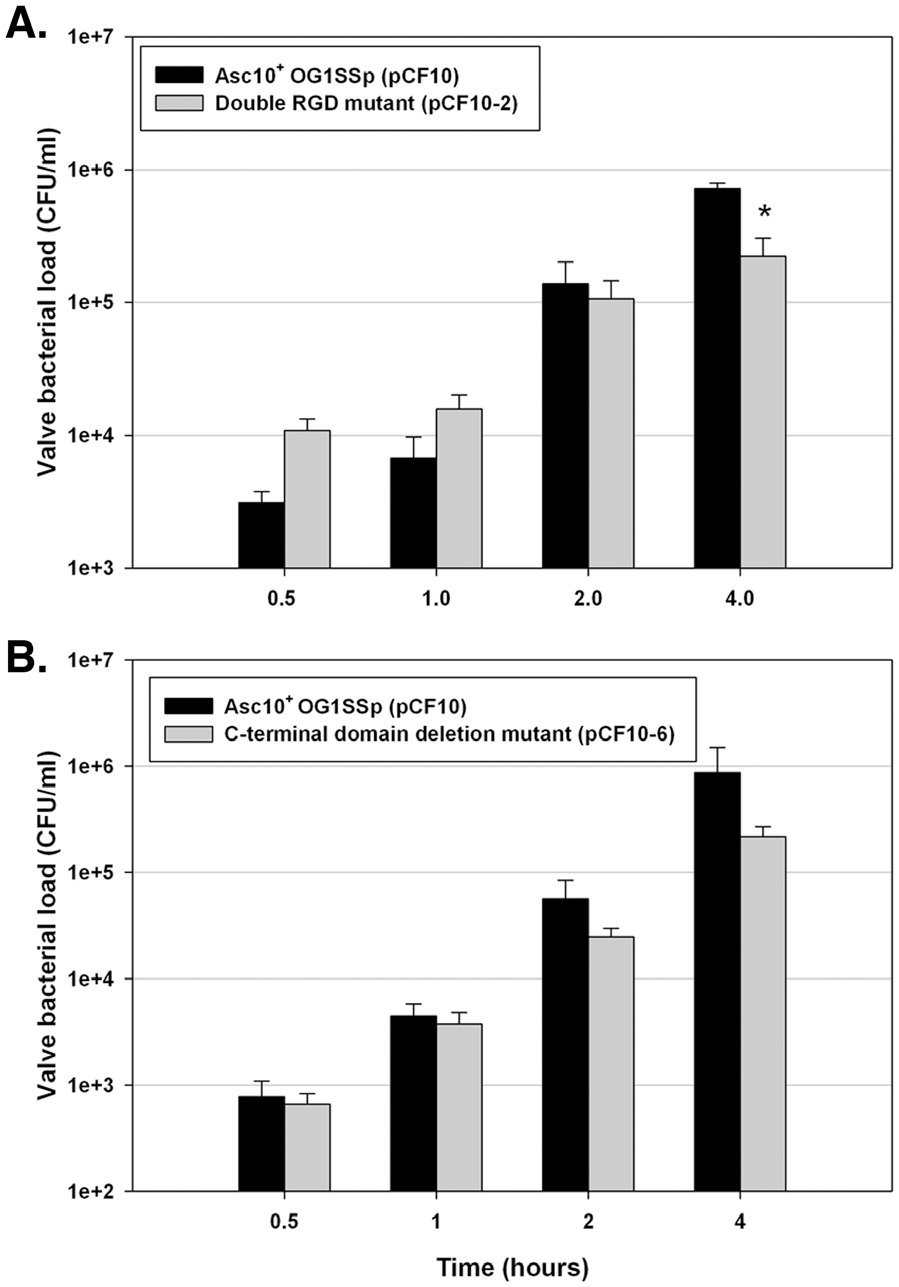
Effects of mutations altering Asc10 subdomains on adherence and biofilm development. (**A**) At initial timepoints, the double RGD mutant (OG1SSp [pCF10-2]) binds as well or better than Asc10^+^ OG1SSp (pCF10); no deficiency in binding to porcine valve tissue was detected until 4 h. (**B**) Deletion of the C-terminal domain of Asc10 does not significantly affect the ability of *E. faecalis* to bind to valve tissue. The data shown are a compilation of at least three experiments. * denotes p≤0.02, with respect to Asc10^+^ OG1SSp (pCF10) valve bacterial loads.

Using combinations of in-frame deletions, in-frame insertions, and directed mutations altering specific codons of *prgB*, we previously identified multiple functional subdomains of Asc10 (**Figure 1**) involved in bacterial aggregation [15] and endocarditis pathogenesis [16]. These domains include two RGD sequence motifs whose mutation to RAD drastically reduced virulence [16] by a mechanism that could involve resistance to phagocytic killing [25], an N-terminal domain and central domain whose disruption reduces adherence and aggregation [26] and a C-terminal region that can be deleted without major effects on aggregation or virulence [15], [16]. We tested the effects of these mutations on development of biofilms using the porcine heart valve model. Mutation of either or both aggregation domains (Fig. 6), or the RGD motifs (Fig. 7A) caused a statistically significant and reproducible decrease of 5–10 fold in adherent bacteria after 4 h of incubation. At earlier time points, no significant differences were observed with the RGD mutations, similar to the results obtained following complete deletion of the protein (Fig. 3D). As seen in previous studies [15], [16], [25], deletion of the C-terminal subdomain did not have a significant impact (Fig. 7B).

## Discussion

Previous studies implicated the plasmid-encoded surface protein Asc10 in enhancing the severity of experimental endocarditis in rabbits [16], [19]. Because biofilm formation likely plays a major role in the production of endocarditis vegetations, we sought to develop a simple model system to examine the heart valve as a surface for enterococcal adherence and biofilm development. Such a model would be useful for distinguishing Asc10 contributions to these steps in endocarditis pathogenesis from other potential functions of the protein, such as modulation of the innate immune response to infection [25], [27]. Also, this model will facilitate studying the role in virulence of other genetic determinants of biofilm formation previously identified using genetic screens [21], [28]. The heart valve tissue system reduces the use of experimental animals since the valves were from animals sacrificed for other purposes and this tissue would have otherwise been discarded. In addition, several strains can be tested simultaneously with valve segments from the same heart, whereas the surgical procedures and biological variability in the rabbit endocarditis model are complex and limit the number of strains that can feasibly be tested. The ex vivo valve model also has limitations, including the absence of a fully functional host defense system. In addition, although the tissue culture plates containing the submerged valve sections were rocked during incubation, the system does not provide the high level of fluid flow and shear forces exerted on valve surfaces in vivo. Future improvements to the system might include the use of flow chambers to better simulate these conditions. Lastly, the 4 h time course of this model does not represent the entire course of an endocarditis infection, which can be a chronic condition. However, the results presented here show its utility for analysis of the initial interactions of the bacteria with the host surface.

In a previous study examining the effects of pCF10 carriage and Asc10 expression on experimental endocarditis in rabbits [16], we found that the bacterial load in endocarditis vegetations produced by a strain with an in-frame deletion of *prgB* was about 10000-fold lower than produced by the strain carrying the wild-type allele. In the present study, we found that both strains attached to the porcine heart valve surfaces and actively initiated biofilm development within 4 h, at levels significantly higher than those observed using abiotic surfaces (Fig. 4). Expression of wild-type Asc10 increased the numbers of adherent bacteria by a factor of 5–10 fold after 4 h (Figs. 3D and 4). The surface distribution of the Asc10^+^ organisms was markedly different, featuring numerous large adherent microcolonies comprised of hundreds of bacteria, and abundant extracellular matrix (Figs. 2C, 3A, 5C, & 5D), as opposed to the smaller sized nascent microcolonies produced by the Asc10^−^ strain at the same time point (Figs. 3C, 5E, 5F). Analysis of strains expressing *prgB* alleles affecting the aggregation and RGD subdomains of Asc10 were also somewhat impaired in biofilm development. The fact that several *prgB* mutations had less severe effects in the in vitro valve model is consistent with previous evidence that part of the contribution of Asc10 to virulence relates to protection of the bacteria from the host innate immune response [25], [27].

**Figure 8:**
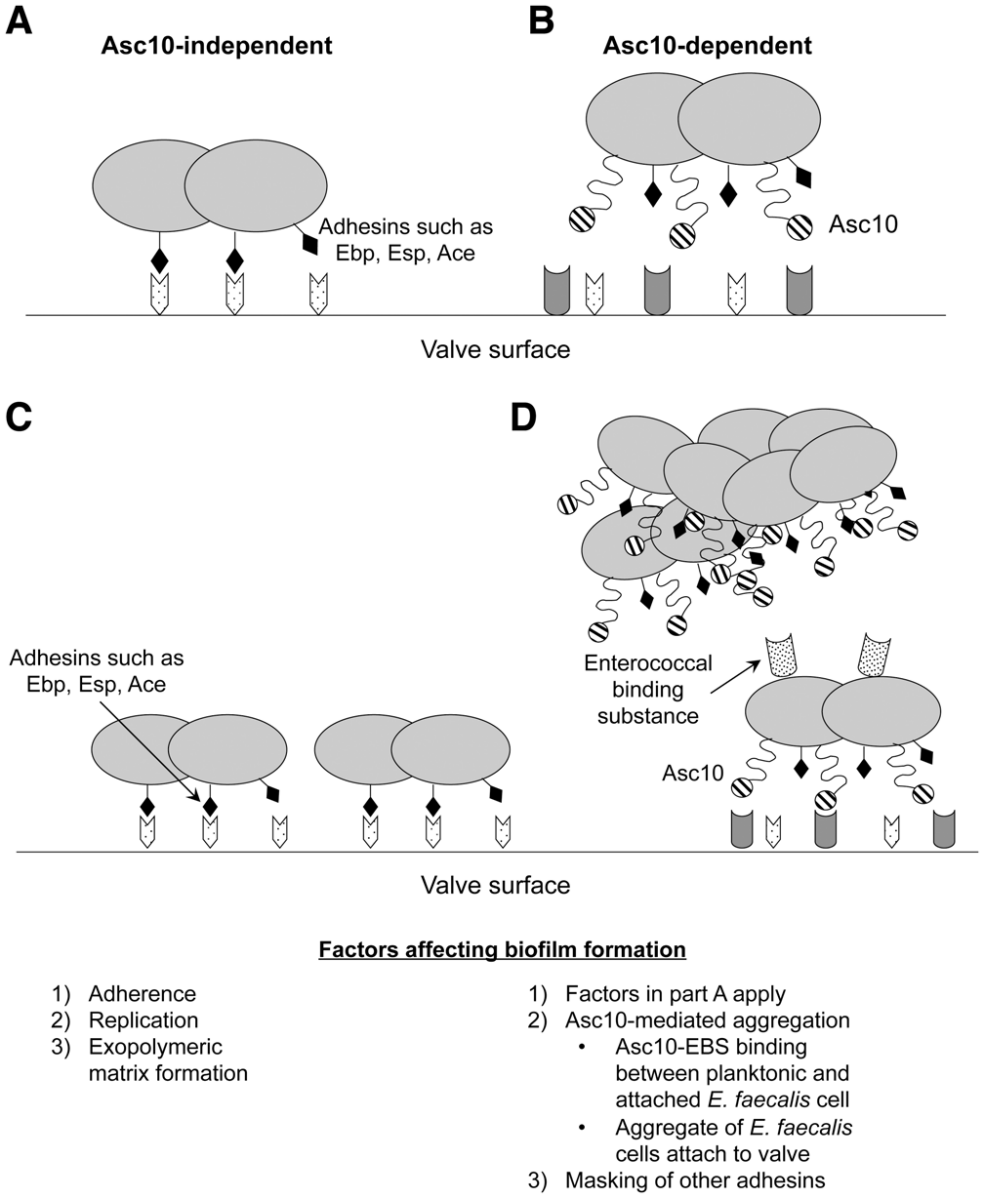
Asc10-independent and -dependent biofilm formation models. *E. faecalis* cell attachment to the valve surface can be mediated by chromosomally-encoded adhesins (**A**), or by Asc10 (**B**). In the absence of Asc10, accumulation of biofilm mass takes place as individual *E. faecalis* cells bind to the valve tissue (**C**). Asc10 expression allows for bacterial aggregation, and thus accelerated growth of biofilm mass through adherence of aggregates to the surface.

The results of this study also suggest the possibility that Asc10 expression in the context of the core enterococcal genome provides *E. faecalis* with two potential pathways of attachment and biofilm formation on host tissues such as heart valves. As illustrated in Fig. 8, multiple adhesins not necessarily associated with pheromone-responsive plasmids have been shown to promote attachment and/or biofilm formation; the Ebp and Ace adhesins have been directly implicated in endocarditis [29], [30]. Asc10 enhancement of attachment to host surface receptors may occur by mechanisms that are functionally redundant with those of other enterococcal adhesins. Thus attachment and initiation of biofilm development can occur with either type of strain. Expression of Asc10 may have two important effects on these processes. One of these is enhancement of the accumulation of adherent microbes by inter-bacterial attachment; this could allow planktonic organisms to bind to the surface via previously attached enterococcal cells, or could lead to surface attachment of many cells from an aggregate formed in the liquid phase via a single adherence event to the host surface. It is interesting that other studies have independently confirmed the importance of aggregation in the enhancement of endocarditis [18]. However, the pCF10-encoded proteins might also reduce surface expression or physically mask expression of other adhesins, such that a strain expressing a defective Asc10 has a significant competitive disadvantage relative to a plasmid-free strain; our previous endocarditis studies strongly suggested this possibility [16]. Asc10 is a very large protein with several functional subdomains, and the aforementioned scenario likely provides selective pressure for maintenance of the fully functional version of the protein that is highly conserved in multiple plasmids [31]. Since attachment and initiation of biofilm development are the initial, critical steps in the pathogenesis of endocarditis, detailed analysis of the molecular aspects of these events, as initiated in this study, might allow for design of therapeutic inhibitory molecules that affect these processes without killing the organisms directly.

## Materials and Methods

### Ethics statement: animal use

Hearts were removed from sacrificed outbred Yorkshire-Landrace pigs (courtesy of Drs. David Brown and Gregory Beilman and the Experimental Surgical Services department at the University of Minnesota; these animals were sacrificed for harvesting of non-cardiac tissues for other research projects). Animals were euthanized with sodium pentobarbital, and all efforts were made to minimize suffering. All animal procedures were carried out according to the established guidelines of the University of Minnesota Institutional Animal Care and Use Committee, and the following protocols were approved by the committee prior to the initiation of these studies (IACUC protocol numbers 0312A54581, 00808A42421, 0803A28761, 0508A72767, 0608A91673, and 0704A06761).

### Bacterial strains and growth conditions

*E. faecalis* strains were grown from frozen glycerol stocks stored at −80°C, and inoculated into Todd-Hewitt (TH; Becton Dickinson, Sparks, MD) broth with the appropriate antibiotics. These cultures were grown at 37°C for 15–16 h. Strains used in this study are listed in Table 1.

**Table 1:** Strains and plasmids used in this study.

Strain or plasmid	Description	Source or reference
**Bacteria**		
*E. faecalis*		
OG1SSp	Streptomycin and spectinomycin-resistant parent strain	[20]
**Plasmids**		
pCF10	Pheromone-inducible conjugative plasmid	[32]
pCF10-1	Asc10 mutant derivative; 31-amino acid insertion in central aggregation domain at nucleotide 1638	[16]
pCF10-2	Asc10 mutant derivative; double RGD mutant with single amino acid substitutions RGD → RAD	[16]
pCF10-4	Asc10 mutant derivative; N-terminal aggregation domain deletion (nucleotides 468-1074)	[16]
pCF10-5	Asc10 mutant derivative; double aggregation domain mutant – 31-amino acid insertion in central aggregation domain, deletion in N-terminal aggregation domain	[16]
pCF10-6	Asc10 mutant derivative; C-terminal domain deletion (nucleotides 2064-3414)	[16]
pCF10-8	In-frame *prgB* deletion mutant retaining first three and last three codons, Asc10^−^	[16]

### Analysis of adherence by field emission scanning electron microscopy (FESEM)

Hearts were removed from pigs being sacrificed for other purposes as described above. The aortic, tricuspid, and mitral valves were removed aseptically using a skin biopsy punch tool (Acuderm; Fort Lauderdale, FL) to divide the valve tissue into 6-mm diameter round sections. Each valve section was placed into separate wells of a 6-well Costar 3516 tissue culture plate (Corning, Inc., Corning, NY). Based on the average weight of the valve sections, a ratio was calculated for each valve section used for SEM studies or for enumeration of adherence bacteria by plating (described in the next section); all volumes of medium, phosphate-buffered saline (PBS) washes, and bacterial inocula were normalized according to this ratio. The standard volumes (prior to normalization) were as follows: endothelial cell medium (ECM; Lonza, Walkersville, MD) for incubation of valves with bacteria  = 4 ml; PBS washes  = 4 ml; bacterial inocululm  = 10 µl; final homogenization buffer for enumeration  = 500 µl. The actual volumes used were the standard volume × the ratio.
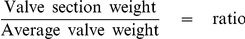



Each valve section was placed in approximately 4 ml ECM supplemented with 5% fetal bovine serum, 500 mg hydrocortisone, 6 mg bovine brain extract, 0.1% human recombinant epidermal growth factor. These valve sections were incubated in ECM plus 100 µg/ml gentamicin at 37°C in 7% CO_2_ for the overnight period with rocking, to ensure lack of contamination. On the following day, the valve sections were washed three times with 4 ml PBS to eliminate any residual gentamicin, with a final replacement by fresh ECM without antibiotics, but with synthetic cCF10 pheromone (1 ng/ml) to ensure expression of *prgB* by the bacteria during the assays. In initial experiments (not shown), we included a brief (60–90 min) treatment of the valve sections with erythromycin (10 µg/ml) immediately after harvesting, then removed the antibiotic and carried out the assays immediately with no antibiotics present. This procedure resulted in occasional bacterial contamination observed on the enumeration plates, so for the experiments reported here, we incorporated the overnight pre-treatment with gentamicin described above. This prevented contamination, and we did not observe significant differences in adherence to uncontaminated valves treated with the short exposure to erythromycin versus the longer exposure to gentamicin.

Bacterial strains were grown overnight without aeration at 37°C in Todd-Hewitt (TH) broth; cells were washed twice and resuspended in PBS at an OD_600_ of 1.5. Approximately 10^6^ CFU/ml of the appropriate bacterial suspension was added to each well containing a valve segment, with subsequent incubation at 37°C in 7% CO_2_ for 0.5, 1, 2, and 4 h, with rocking.

Valve sections incubated with bacteria were washed three times in approximately 4 ml PBS by flicking of tubes for removal of loosely associated bacteria. Each valve was then placed in approximately 4 ml of FESEM buffer (7% sucrose, 100 mM sodium cacodylate buffer, and 3% glutaraldehyde) with incubation at room temperature overnight for fixation. For some valve sections, addition of the cationic dye alcian blue (0.15%) to the FESEM buffer was used to stabilize the biofilm matrix for visualization. Valve tissue was stored in fixative at 4°C for 2 days, then washed in 100 mM sodium cacodylate buffer and postfixed in 1% osmium tetroxide and 1.5% potassium ferricyanide in cacodylate buffer. All FESEM buffers and aldehyde solutions were obtained from Electron Microscopy Services (Hatfield, PA). The samples were rinsed, dehydrated through a graded ethanol series, with subsequent critical point drying with CO_2_. A coating of 1 to 2 nm platinum was applied to the valve sections with an argon ion beam coater (Denton DV-502), and imaging was done on a Hitachi S-4700 field emission scanning electron microscope (FESEM) operated at 2 to 3 kV. Quartz PCI software was utilized to obtain images, where they were stored in a TIFF format.

### Enumeration of bacteria from adherence assays

Tricuspid, aortic, and mitral valves were removed from porcine hearts as described in the previous section. Valve sections were infected with bacteria as described above, and at each time point (0.5, 1, 2, and 4 h), valves were washed as before. The washed valves were then placed in about 500 µl PBS and treated with a motorized pestle (Fisher Scientific, Hanover Park, IL) for 2 min to remove adherent bacteria. To quantify bacteria, aliquots from all washes and medium after incubation with bacteria were serially diluted and plated onto TH agar with the appropriate antibiotics (streptomycin 1000 µg/ml, spectinomycin 250 µg/ml, erythromycin 10 µg/ml).

Results are expressed as a compilation of the actual CFU/ml values from all experiments, since the volume of buffer used for resuspending adherent bacteria was normalized to the weight of each valve segment. Values for each strain's valve-adherent bacterial loads (CFU/ml) were averaged in generating each plot.

Adherence assays were also performed with circular cellulose membranes (1-cm diameter), with use of the Troemner Multitube vortexer (Thorofare, NJ) to remove adherent bacteria. Results are reported as CFU/mm^2^ because a CFU/surface mass comparison between the cellulose membrane and valve tissue results was not relevant. Therefore, surface area was calculated for each system, where the height of the valve tissue was assumed to be 1 mm, and height for the cellulose membranes was considered too low to contribute significantly to the surface area.

### Statistical analysis

Microbial loads (CFU/ml) were analyzed using an unpaired Student's t-test; one-way analysis of variance followed by Fisher's post-hoc testing was utilized to analyze the aggregation domain mutant data in comparison to Asc10^+^ OG1SSp (pCF10). Data from Figure 4 were analyzed using an unpaired Student's t-test. Statistical significance was p<0.02 for all data analyzed.

## Supporting Information

Figure S1 **Use of different techniques to disperse Asc10^+^ OG1SSp (pCF10) aggregates in broth culture.** Asc10^+^ OG1SSp (pCF10) and the *prgB* deletion mutant were grown in endothelial cell medium for 3 h. Aliquots of the broth culture were treated with the following conditions: (1) 1 min of sonication plus a 1.5 min treatment with a motorized pestle, (2) 2 min motorized pestle alone, (3) 2 min motorized pestle with addition of 2 mM EDTA. Bacterial loads were quantified from the medium at the start of the experiment and after 3 h before any treatment, as well as from each of the dispersal techniques. Essentially the ability of each treatment to disperse aggregates of the *E. faecalis* cells was fairly equal. In our study, we used the pestle treatment for 2 min, and thus this data demonstrates that the Asc10^+^ OG1SSp (pCF10) aggregates are broken up by pestle treatment sufficiently.
(PDF)
