# Supplementary material for: Acceleration of Enterococcus faecalis Biofilm Formation by Aggregation Substance Expression in an Ex Vivo Model of Cardiac Valve Colonization
Source: PLoS One. 2010 Dec 30;5(12):e15798. doi: 10.1371/journal.pone.0015798 (PMC3012704; doi:10.1371/journal.pone.0015798)
Supplement: Figure S1 — Use of different techniques to disperse Asc10+ OG1SSp (pCF10) aggregates in broth culture. Asc10+ OG1SSp (pCF10) and the prgB deletion mutant were grown in endothelial cell medium for 3 h. Aliquots of the broth culture were treated with the following conditions: (1) 1 min of sonication plus a 1.5 min treatment with a motorized pestle, (2) 2 min motorized pestle alone, (3) 2 min motorized pestle with addition of 2 mM EDTA. Bacterial loads were quantified from the medium at the start of the experiment and after 3 h before any treatment, as well as from each of the dispersal techniques. Essentially the ability of each treatment to disperse aggregates of the E. faecalis cells was fairly equal. In our study, we used the pestle treatment for 2 min, and thus this data demonstrates that the Asc10+ OG1SSp (pCF10) aggregates are broken up by pestle treatment sufficiently. (PDF) [file pone.0015798.s001.pdf]

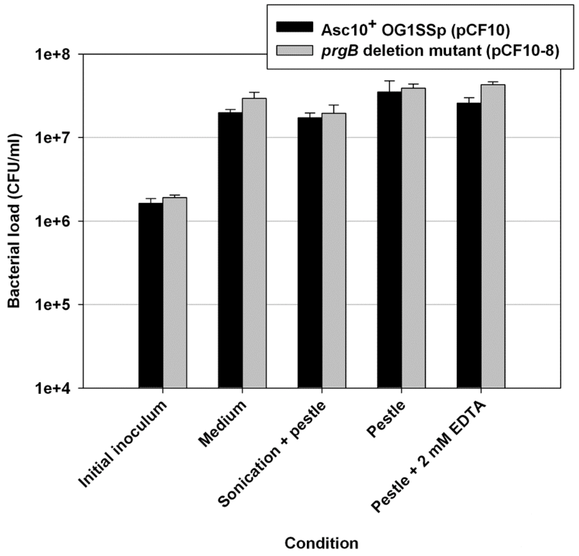

Supplemental figure 1. Use of different techniques to disperse Asc10$^+$ OG1SSp (pCF10) aggregates in broth culture. Asc10$^+$ OG1SSp (pCF10) and the *prgB* deletion mutant were grown in endothelial cell medium for 3 h. Aliquots of the broth culture were treated with the following conditions: (1) 1 min of sonication plus a 1.5 min treatment with a motorized pestle, (2) 2 min motorized pestle alone, (3) 2 min motorized pestle with addition of 2 mM EDTA. Bacterial loads were quantified from the medium at the start of the experiment and after 3 h before any treatment, as well as from each of the dispersal techniques.

Essentially the ability of each treatment to disperse aggregates of the *E. faecalis* cells was fairly equal. In our study, we used the pestle treatment for 2 min, and thus this data demonstrates that the Asc10$^+$ OG1SSp (pCF10) aggregates are broken up by pestle treatment sufficiently.
